# Supplementary material for: LncRNA MHRT Prevents Angiotensin II-Induced Myocardial Oxidative Stress and NLRP3 Inflammasome via Nrf2 Activation
Source: Antioxidants (Basel). 2023 Mar 9;12(3):672. doi: 10.3390/antiox12030672 (PMC10044972; doi:10.3390/antiox12030672)
Supplement: Supplementary file 1 [file antioxidants-12-00672-s001.zip › antioxidants-2127743-supplementary.pdf]

# Supplementary Materials

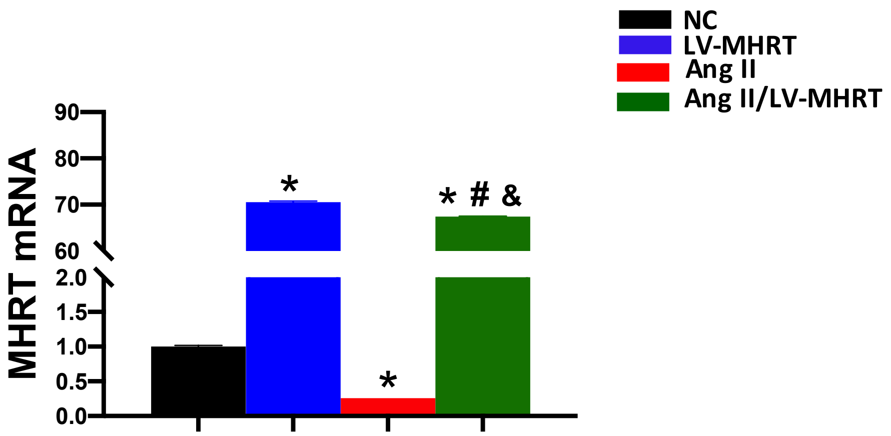


**Figure S1.** The expression of MHRT in AC16 cardiomyocyte with MHRT overexpression and Ang II treatment. AC16 cells of LV-MHRT and NC were stimulated with/without Ang II (100nM) for 24h and mRNA was harvested to analyze the transcription of MHRT by qPCR. Data are presented as the mean ± SD (n = 6). * $p$ <0.05 vs. NC, #$p$ <0.05 vs. Ang II, &$p$ <0.05 vs. LV-MHRT.

**Table S1. The primers for human AC16 cells**

| Gene | Forward Primer | Reverse Primer | Gene Accession Number |
|---|---|---|---|
| GAPDH | GGTGAAGCAGGCGTCGGAGG | GAGGGCAATGCCAGCCCAG | NM_001289746.2 |
| MHRT | CCGACTGCGACTCCTCATAC | GGCTGAAGAGTGAGCCTTGT | NR_126491.1 |
| NLRP3 | GTGGAGATCCTAGGTTTCTCTG | CAGGATCTCATTCTCTTGGATC | NM_001243133.2 |
| caspase-1 | ACACGTCTTGCCCTCATTATCT | ATAACCTTGGGCTTGTCTTTCA | NM_001257119.3 |
| IL-1β | CCCTGCAGCTGGAGAGTGTGG | TGTGCTCTGCTTGAGAGGTGC | NM_000576.3 |
| Nrf2 | CTTGGCCTCAGTGATTCTGAAGTG | CCTGAGATGGTGACAAGGGTTCA | NM_001313904.1 |
| CAT | TGAAGATGCGGCGAGACTTT | TGGATGTAAAAAGTCCAGGAGGG | NM_001752.4 |
| HO-1 | ATGGCCTCCCTGTACCACATC | TGTTGCGCTCAATCTCCTCCT | NM_002133.3 |
| TXNIP | CTTGCGGAGTGGCTAAAGTG | ATTCTCACCTGTTGGCTGGT | NM_006472.6 |

**Table S2. The primers for mouse tissues**

| Gene | Forward Primer | Reverse Primer | Gene Accession Number |
|---|---|---|---|
| GAPDH | GCAAGTTCAACGGCACAG | GCCAGTAGACTCCACGACAT | NM_001411845.1 |
| MHRT | GAGCATTTGGGGATCCTATAC | TCTGCTTCATTGCCTCTGTTT | NR_033497.1 |
| Nrf2 | TCACACGAGATGAGCTTAGGGCAA | TACAGTTCTGGGCGGCGACTTTAT | NM_010902.5 |
| NLRP3 | AGAAGAGACCACGGCAGAAG | CCTTGGACCAGGTTCAGTGT | NM_145827.4 |
| caspase-1 | TCAGCTCCATCAGCTGAAAC | TGGAAATGTGCCATCTTCTTT | NM_009807.2 |
| IL-1β | TTCCTTGTGCAAGTGTCTGAAG | CACTGTCAAAAGGTGGCATTT | NM_008361.4 |
| HO-1 | AGGTACACATCCAAGCCGAGA | CATCACCAGCTTAAAGCCTTCT | NM_010442.2 |
| CAT | GGAGGCGGGAACCCAATAG | GTGTGCCATCTCGTCAGTGAA | NM_009804.2 |
